# Pediatric Liver Transplantation

**DOI:** 10.1155/1991/73649

**Published:** 1991

**Authors:** Philip Seu, Ronald W. Busuttil

**Affiliations:** M.D., Ph.D. Room 77-132 CHS UCLA Medical Center Los Angeles CA 90024 USA


*HPB Surgery*, 1991, Vol. 3, pp. 145-166
Reprints available directly from the publisher
Photocopying permitted by license only

© 1991 Harwood Academic Publishers GmbH
Printed in the United Kingdom

### PEDIATRIC LIVER TRANSPLANTATION

PHILIP SEU and RONALD W. BUSUTTIL

*(Received 23 April 1990)*

### INTRODUCTION

Liver transplantation is the treatment of choice for various causes of end-stage liver disease in children[1]. Remarkable progress has been made in the field since the first human orthotopic liver transplant was performed in a child in 1963[2]. The number of transplants performed has increased especially since 1983, when liver transplantation was given therapeutic status by the NIH consensus conference[1]. By October 1986, five U.S. centers had performed more than 20 pediatric transplants each, but in 1988 it is estimated that approximately 280 pediatric OLT's were performed. The majority are done in eight major centers and this concentration of experience is necessary for continued optimal results.

This overview of the field will attempt to highlight aspects of liver transplantation especially pertinent to pediatrics including childhood diseases, technical considerations in children, and technical complications.

### DISEASES TRANSPLANTED

The diseases in pediatric patients treated with OLT at UCLA are listed in Table 1. Biliary atresia was the most common followed by inborn errors of metabolism, and "cirrhosis" from various causes. This is consistent with reports from other institutions with large pediatric OLT programs[3].

### BILIARY ATRESIA

This is the most common indication for liver transplantation in children as noted above. Biliary atresia can be defined as a partial or complete absence of patent bile ducts. The incidence varies from 1:8,000 to 1:10,000 with a 4-5 fold increase in Pacific and Indian Ocean areas[4]. In approximately 2–3% of cases of extrahepatic biliary atresia(EHBA), surgical exploration reveals a dilated hilar structure which may communicate with intrahepatic bile ducts, the so-called "correctable" type of EHBA. Before 1959, the natural history of patients with "uncorrectable" biliary atresia was progressive liver failure and death before age two months. In 1959, Kasai and Suzuki devised the operation of hepatoenterostomy which consisted of using a defunctionalized loop of jejunum to drain microscopic ducts within the porta

Address correspondence to: Ronald W. Busuttil, M.D., Ph.D., Room 77–132 CHS, UCLA Medical Center, Los Angeles, CA 90024.

**Table 1** Pediatric liver transplants

| *Disease* | *Patients transplanted* | *Deaths* |
|---|---|---|
| Biliary atresia | | |
| Biliary atresia | 76 | 19 |
| Biliary hypoplasia | 2 | |
| Alagille's syndrom | 2 | |
| Nonsymptomatic paucity of intrahepatic bile ducts | 1 | |
| Metabolic disorders | | |
| Alpha-1-antitrypsin deficiency | 13 | 2 |
| Tyrosinemia | 2 | 1 |
| Sanfilippo syndrome | 1 | |
| Byler's syndrome | 1 | |
| Gaucher's syndrome | 1 | 1 |
| Cirrhosis | | |
| Idiopathic cholestasis/cirrhosis | 9 | |
| Familial cirrhosis | 7 | 1 |
| Chronic active hepatitis | 7 | 2 |
| Inflammatory | | |
| Acute hepatic necrosis | 9 | 2 |
| Subacute hepatic necrosis | 1 | |
| Tumors | | |
| Hepatoblastoma | 1 | 1 |
| Hemangioendothelioma | 1 | |
| Metastatic leiomyosarcoma | 1 | |
| Hepatocellular carcinoma | 1 | 1 |
| Miscellaneous | | |
| Budd-Chiari syndrome | 1 | |
| Total | 137 | 30 |

hepatis[5]. Since then, numerous modifications of the procedure have occurred[6]. The operation needs to be performed early in life, generally within four months. Successful bile flow is achieved in 40–60% of patients operated on before age four months[7,8]. Even with initially successful bile drainage, many patients will experience progressive cholestasis and cirrhosis, possibly due to an intrahepatic cause[9]. The most common clinically identifiable cause of failure of an initially succesful Kasai procedure is cholangitis[10]. Other causes of late failure include inadequately sized periportal bile ducts and progression to fibrosis. Ninety percent of children in whom a Kasai procedure fails to provide adequate permanent bile drainage will die by age 5 years[11]. Reattempts at bile drainage after an initially unsuccessful Kasai procedure have been uniformly poor[12,13].

In a group of 31 patients transplanted for biliary atresia at Pittsburgh, early survival was 84% (followup of 2–36 months) and prior biliary surgery did not significantly affect survival after OLT[12]. In the UCLA experience reported in 1988, 28 of 36 patients who underwent OLT for biliary atresia had undergone prior hepatic portoenterostomy. This group had no significant differences in intraoperative blood loss, biliary complications or six month survival from the group without prior Kasai procedures. The overall three year actuarial survival was 75%[14]. To date, 76 patients have received OLT for biliary atresia at UCLA with an overall survival of 75%.

In conclusion, children with biliary atresia should undergo early portoenterostomy, but at the first sign of failure should be referred for OLT evaluation.

### INBORN ERRORS OF METABOLISM

### *Alpha-1-antitrypsin deficiency*

This is the most common of the metabolic disorders for which OLT is performed. Cirrhosis associated with ATT deficiency was first reported in 10 children in 1969[15]. Alpha-1-antitrypsin is one of several serine proteases synthesized in the liver which inhibit a wide variety of proteolytic enzymes within plasma, including trypsin[16]. The gene for the enzyme has 24 alleles with autosomal co-dominance. The Pi(protease inhibitor) MM phenotype is normal. Most end-stage liver disease is associated with the PiZZ phenotype and they usualy have between 10–15% of the normal serum ATT level of 200–400mg/dl[17]. They are also susceptible to emphysema which generally develops in adults. The incidence of PiZZ among 200,000 infants screened was 125(.06%). Of these, 14(11%) had neonatal cholestatic jaundice, and at age two years, three of the 14(27%) progressed to cirrhosis. The overall development of cirrhosis being 3 out of 200,000 infants[18].

The pathogenesis of hepatic injury is felt to be due to either hepatocyte damage by uninhibited proteolysis, and/or accumulation of ATT deposits within hepatocytes[19]. Clinical cholestasis usually develops before ten weeks of age. Neonatal cholestasis usually abates until late childhood or early adolescence when cirrhosis and portal hypertension appear. The progression of cirrhosis and liver failure is variable. The diagnosis should be considered in any neonate with jaundice. Absent alpha-1 globulin peak in serum electrophoresis increases suspicion and can be confirmed by low serum ATT activity, quantitative measures of serum AT, and genetic Pi typing[20]. OLT is offered to patients with cirrhosis and evidence of progressive hepatic decompensation. Following successful OLT, recipients acquire the phenotype of the donor and ATT levels normalize[21] and progression of pulmonary disease is presumably eliminated by OLT. Five year actuarial survival of children transplanted for ATT has been reported to be up to greater than 80%[22].

### *Tyrosinemia*

This is a hereditary disorder characterized by a deficiency of p-hydroxyphenol pyruvate hydroxylase, an enzyme which degrades metabolic products of tyrosine metabolism[23]. The hepatic disease ranges from early onset of acute hepatic failure to slowly progressive cirrhosis. Patients also have a significantly increased incidence of hepatocellular carcinoma. Four of nine patients in one series who successfully underwent OLT for tyrosinemia had malignancy at the time of referral[24].

### *Wilson's Disease*

Wilson's disease is an autosomal recessive disorder characterized by accumulation of copper in the liver, central nervous system, kidney, eyes, and other organs. The prevalence in 1/30,000 worldwide with a carrier frequency of 1/90[25]. The metabolic defect is in the excretion of copper from hepatocellular lysozymes which results in accumulation and subsequent hepatic damage. When the hepatic capacity is exceeded, copper diffuses into the blood and other organs. Characteristic Kayser-Fleischer rings in patient's corneas are copper deposits in Descement's membrane[26].

The clinical course of hepatic manifestations is variable. It may have an insidious onset of progressive hepatic insufficiency and resultant synthetic dysfunction and portal hypertension, or the hepatic disease may be acute and self-limited, or it may be fulminant. The fulminant form occurs most commonly in adolescent patients[26].

The diagnosis is generally made by measuring low serum ceruloplasmin concentrations (<20mg/dl.), with the presence of Kayser-Fleischer rings in the appropriate clinical setting. Liver biopsy with quantitative copper measurements and increased urinary copper excretion confirm the diagnosis[26].

The initial treatment is copper chelation with d-penicillamine. If started before irreversible tissue damage occurs, medical treatment is usually effective[27]. In contrast, such treatment is ineffective when cases present as fulminant or severe subacute hepatitis. The fulminant form of Wilson's disease usually differs from other usual causes of fulminant hepatitis by the presence of a profound Coomb's negative hemolytic anemia, moderate elevation of transaminases and alkaline phosphatase levels while having a very high bilirubin and profound hepatic failure[28].

The indications for OLT in Wilson's disease include fulminant hepatitis, cirrhotics with hepatic decompensation who failed to improve with two to three months of D-penicillamine treatment, and patients who have been effectively treated, then fail after stopping the D-penicillamine[26]. Patients do well after successful OLT, and plasma levels of coper and ceruloplasmin normalize[3].

*Crigler-Najjar Syndrome*

This is a syndrome described in 1952 by Crigler and Najjar as "congenital familial nonhemolytic jaundice"[29]. It has been divided into Type I and Type II. Type I is characterized by undetectable bilirubin glucuronyl transferase activity in liver tissue and unresponsiveness to phenobarbital therapy. These patients progress to death from kernicterus usually before age 15 months[30] and should be considered early for OLT. Type II disease, on the other hand, is less severe and often responds to phenobarbital therapy.

*Protoporphyria*

This is an autosomal dominant disease characterized by elevated levels of protoporphyrin in erythrocytes, plasma and feces. The biochemical abnormality is in deficiency of heme synthase (ferrochelatase) activity[31]. Photosensitivity is the main clinical manifestation but progressive liver damage may occur due to hepatic deposits of protoporphyrin crystals. Medical treatment of the disease is with cholestyramine[30], and OLT has successfully treated refractory cases[3].

Other metabolic diseases treated with OLT include glycogen storage diseases, hemochromatosis, enzymatic deficiency of the urea cycle, and homozygous type II hyperlipoproteinemia[32].

### ACUTE FULMINANT LIVER FAILURE

This syndrome is defined as the development of liver necrosis and hepatic encephalopathy within eight weeks of onset of symptoms in a patient without a prior history of liver disease[33]. The etiologies are diverse and include viral hepatitis, exposure to toxins, Wilson's disease, Budd-Chiari syndrome, and pregnancy. The clinical course is one of rapid deterioration of liver function and onset of neurological failure often progressing to coma and death. The mortality rate is reported up to 60–85% despite maximal supportive medical treatment[34,35,36]. Liver transplantation for fulminant liver failure was first performed in 1968 and since then, progressively more have been done with an over 60% survival[37].

In evaluating fulminant patients for OLT, a thorough neurological evaluation is necessary to assess for reversibility of neurologic deficits. Nine pediatric patients have been transplanted at UCLA for acute hepatic necrosis with a 78% current survival.

### BUDD-CHIARI SYNDROME

The Budd Chiari syndrome results from obstruction of the hepatic veins[38]. Etiologies in the U.S. include hypercoagulable states secondary to various causes including systemic lupus erythematous, polycythemia, and oral contraceptive use. Mechanical causes include membranous obstruction or external compression from malignancy.

Presentation is usually in a young person who develops rapid onset of abdominal pain, ascites, and hepatomegaly which may be associated with liver failure. The diagnosis is confirmed via hepatic venous and inferior vena caval angiography at which time venous pressures should be measured.

Therapy is portosystemic shunting and anticoagulation if hepatic failure is not present. A mesocaval H-graft is recommended unless a gradient exists in the vena cava in which case a mesoatrial shunt is preferred[39]. OLT is offered if hepatic failure is associated with the Budd-Chiari syndrome and a 71% 1 year survival has been reported[40]. At UCLA, we have transplanted four females (average age 25 years) for Budd-Chiari with three patients currently alive. Post-transplant anticoagulation is recommended if hypercoagulability is the underlying etiology. The Cambridge group[41] however feel that it is difficult to distinguish Budd-Chiari patients who are likely to develop rethrombosis and they report 16 patients with liver failure and Budd-Chiari who were all routinely anticoagulated after OLT. No recurrrent hepatic vein thrombosis occurred during a mean followup of 28 months. The post-operative hemorrhagic complication rate was $<14\%$ and none were lethal. The overall three year survival was 88%.

### CHRONIC ACTIVE HEPATITIS

Chronic active hepatitis (CAH) is a pathologic diagnosis in which there are chronic inflammatory and fibrosing hepatic lesions that is often progressive to cirrhosis and liver failure[42]. Etiologies are varied and include viral hepatitis (A,B, or NANB), or rarely hepatic injury from toxins, alcohol or "idiopathic" causes. Hepatitis B is the most common cause and HbsAg is a marker of the disease. Patients with CAH and cirrhosis develop the usual manifestations of end stage liver disease, and is one of the more common indications for OLT. The issue of recurrent disease after OLT for hepatitis B induced CAH is an important one. The presence of HBeAg indicates viral replication and these patients may have an increased likelihood of reinfection of the transplanted graft. The incidence of recurrent B antigen positivity after OLT is reported to be between 50-100%[43,44]. In a review of 31 patients transplanted with hepatitis B at UCLA, 11 (35%) had clinical or pathologic evidence of recurrent hepatitis. Among these 11, seven have died from the disease. The mean time from OLT to death from recurrent hepatitis was 9.6 months. (range 5–19 months).

### MALIGNANCY

Patients with tumors confined to the liver would appear to be excellent candidates for OLT since they do not suffer from all the disabling manifestations of chronic liver disease. Short term survival has beeen excellent in these patients, but approximately 50% eventually die from recurrent disease[45]. Analysis of our experience at UCLA reveal a five year actuarial survival of 62.3% for malignancies excluding hepatocellular carcinoma (HCC), but 16.3% for HCC. Concerning childhood malignancies, a pooled review of 12 children undergoing OLT for hepatoblastoma in the U.S. showed that 50% were still alive at 24 to 70 months after OLT without evidence of disease[46]. One-half of the deaths in the series were from tumor recurrence. Besides a careful preoperative search for extrahepatic spread in all cases, staging exploratory celiotomy is recommended for HCC. Despite all efforts, instances of finding extrahepatic tumor at the time of recipient hepatectomy is possible, and having a second recipient available is recommended.

### PATIENT EVALUATION AND SELECTION

The proper selection of patients for transplantation is crucial to a liver transplant program. A multidisciplinary approach is used and inpatient evaluation is performed by a team that includes transplant surgeons, pediatric hepatologists, nephrologists, psychiatrists, anesthesiologists and infectious disease specialists. During the first 57 months of liver transplantation at UCLA, 225 children were evaluated for OLT. Among these, 37% were less than 12 months of age and 60% were females. Of those evaluated, 69% were accepted and placed on an active waiting list for OLT. Indications for OLT was the presence of any of the following without a contraindication:1) biliary atresia with failed portoenterostomy, 2) metabolic liver disease or cirrhosis leading to liver failure as evidenced by bleeding varices or synthetic dysfunction, 3) fulminant or subacute progressive hepatic failure, 4) cholestatic disorders resulting in persistent jaundice, pruritis, fatigue, growth retardation, or the inability to attend school, and 5) primary liver tumors confined to the liver[47].

Routine blood chemistries and various cultures are performed. In addition, portal vein ultrasonography is used to assess patency. CT scanning is used to evaluate for the presence of extrahepatic malignancy when the possibility exists. Contraindications to OLT include systemic diseases not curable by OLT, irreversible neurologic changes, extrahepatic malignancy, or sepsis outside the biliary tract. Patients are accepted or rejected as candidates by committee, and an assessment of urgency is assigned. Donor to recipient matching is based on ABO status and size, and priority is given to more urgent candidates. Donor body weights between 2.5 to

.5× that of the recipient have been used. Transplantation across ABO barriers have been done in urgent situations. (See ABO mismatched graft.)

### TECHNICAL CONSIDERATIONS

The recipient hepatectomy is the most difficult part of the transplant procedure. Prior operations especially in biliary atresia patients, coagulopathy, and portal hypertension all contribute to the increased difficulty. Almost all children with biliary atresia have undergone a portoenterostomy prior to coming to transplantation. Many have had multiple attempted revisions and have dense scar tissue in the operated field. In such patients dissection of the portal structures is begun with the posterior aspect of the right lobe of the liver, in a previously unviolated plane. The right hepatic lobe can be reflected away from the duodenum and transverse colon, and the Roux-en-Y limb identified and traced to the hepatic hilum. Next, access to the portal structures is gained by transecting the Roux-en-Y limb and reflecting it.

After the portal structures are dissected, the supra- and infrahepatic vena cavae are isolated. In contrast to adults in whom veno-venous bypass is routinely used, infants and small children tolerate venous occlusion fairly well, and veno-venous bypass is rarely needed. Before the native liver is removed, trial clamping of the vena cava is performed. Some of the larger children will not tolerate the clamping and require bypass. After the recipient hepatectomy is completed, perfect hemostasis must be achieved in the hepatic fossa, and this requires reapproximation of the open peritoneal surfaces over the bare area and both triangular ligaments.

The graft implantation in children is especially challenging due to the small caliber and delicate nature of the anastomoses. The anastomoses are performed in the following order: suprahepatic inferior vena cava, infrahepatic inferior vena cava, portal vein, hepatic artery, and biliary system. The vena caval anastomosis is performed with 4–0 or 5–0 polypropylene interrupted anterior row and running posterior row. The interrupted sutures facilitate growth of the anastomosis. The portal vein anastomosis is a running 6–0 or 7–0 polypropylene suture unless the vessel is too small in which case interrupted sutures are used. The new graft is then perfused with portal blood while the arterial anastomosis is performed. The method of arterial reconstruction depends on the anatomy of the donor and recipient and an adaptive intraoperative strategy is necessary. The source of inflow must be adequate to avoid technical failure. The preferred reconstruction is an aortic carrell patch of the donor to a branch patch[48] of the celiac aorta of the recipient. A running 6–0 or 7–0 polypropylene suture is usually used. The biliary reconstruction in children is usually a Roux-en-Y choledochojejunostomy over an internal stent to a 40 cm defunctionalized limb of jejunum. If the duct is not diseased and is of sufficient caliber, an end-to-end choledochocholedochostomy over a T-tube is preferred, but rarely possible in the pediatric population. After the graft is implanted, three closed suction drains are placed before closure of the abdomen.

### REDUCED-SIZE LIVER TRANSPLANTATION

The transplantation of part of a liver was initially done in heterotopic liver transpslantation due to size constraints and first reported by Fortner[49]. With increases in centers performing OLT, a shortage of smaller donors exists. The attrition rate of pediatric patients on waiting lists is reported to be up to 25–30%[50]. At UCLA, the figure is 10% and usually death is due to lack of a suitable donor. Orthotopic transplantation of a reduced-size graft was initially reported by Bismuth in Europe where the shortage of organs is even greater than in the U.S[51]. Recently, Broelsch reported his experience with 14 children who received reduced liver grafts[52]. Thirteen of the 14 were urgent cases. These included three right lobe grafts, nine left lobes, and two left lateral segments. Patient survival was 50% which the authors felt were comparable to similar high-risk recipients of full-sized grafts. The graft related and extrahepatic complication rates were 71% and 93%, respectively, in the 14 patients. Guidelines by Broelsch include two independent surgical teams when performing reduced-liver transplantation, use of donors up to 4–6 × recipient weight, and determination of which lobe to use after opening the recipient's abdomen.

The largest experience to date with reduced-size grafting was reported by Otte, *et al*[53]. They report 54 reduced-size grafts of which 30 were performed electively. The overall one year patient survival was 82% for full grafts vs. 68% for reduced-sized grafts. The one year survival of the 30 children who "electively" received reduced grafts was 77%.

The complication rates after reduced-size grafting remains higher than that of full-size grafts, but reduced-liver transplantation should be considered in emergency situations when a size-matched donor is not available, and the surgeon has the necessary technical experience.

### LIVER PRESERVATION

One of the more exciting recent developments in liver transplantation has been that of extended liver preservation using a solution developed by Belzer at the University of Wisconsin.(UW solution) Initial clinical trials demonstrated good function in 17 livers stored in UW. In 9 of these, mean preservation times was 12.7 hours (range 11–20)[54]. The solution evolved from Belzer's earlier work when he showed the importance of phosphate and adenosine in kidney preservation[55]. In addition to these compounds, UW solution contains unique polymer sugars (hydroxyethyl starch, lactobionate) as nonionic osmotic agents, as well as glutathione and allopurinol as antioxidants and oxygen free radical scavengers.

At UCLA we started using UW solution for liver preservation in May of 1988. This has permitted significantly longer preservation times without compromise of graft function. It is felt that the ability to lengthen preservation times will help change the nature of liver transplantation practice as it permits greater flexibility in utilization of operating room time and resources. Also the timing of donor and recipient operation is not as critical, and this permits improved utilization of available donor organs.

### COMPLICATIONS

The complications of orthotopic liver transplantation to be discussed are outlined on Table 2. They have been somewhat arbitrarily divided into "hepatic" and "nonhepatic".

**Table 2** Complications of orthotopic liver transplantation

| I Hepatic | II Nonhepatic |
|---|---|
| A Technical | A Infection |
| 1 Harvest injuries | B Renal |
| 2 Arterial | 1 hepatorenal |
| hepatic artery thrombosis | 2 acute tubular necrosis |
| pseudoaneurysms | 3 drug toxicity |
| 3 Venous | 4 dialysis |
| hypoplasia/absence | C Gastrointestinal |
| thrombosis | 1 bleeding |
| 4 Biliary | 2 perforation |
| leak | 3 obstruction |
| obstruction | 4 pancreatitis |
| B Nontechnical | D Pulmonary |
| 1 Primary nonfunction | 1 atelectasis |
| 2 Rejection | 2 effusion |
| acute | 3 respiratory failure/ARDS |
| chronic | 4 infection |
| 3 Mismatched graft | E Neurological |
| | 1 seizures |
| | 2 hemorrhage |
| | 3 positioning injuries |
| | F Drug toxicity |

### HARVEST COMPLICATIONS

Technical harvest complications include injury to unrecognized aberrant right or left hepatic arteries, liver laceration, or bowel injury. With multiorgan harvests from the same donor, instances of cutting the suprahepatic vena cava too short for optimal liver implantation have occurred. Other complications include hemodynamically unstable donors and unrecognized pre-existing disease in the donor liver. The consequences of these complications have ranged from the need to repair an injury to cancellation of a transplant operation.

### HEPATIC ARTERY THROMBOSIS

This is one of the most devastating complications following OLT. The incidence of hepatic artery thrombosis (HAT) is significantly higher in pediatric series with rates of 7.4–33% reported[56,57,58].

The presentation of hepatic artery thrombosis can be categorized into 3 general syndromes[58]. 1.) The first is that of fulminant hepatic necrosis and sepsis. Patients develop rapid onset of hepatic decompensation and sepsis with fever, altered mental status, hypotension, and coagulopathy. Laboratory studies reveal markedly elevated liver enzymes, leukocytosis and prolonged protimes. Blood cultures are frequently positive for gram negative enterics and also anaerobes. Radiographs may show gas gangrene of the liver. 2.) The second presentation is that of relapsing bacteremia frequently with hepatic abscesses. Patients develop fever, mild increases in liver enzymes, leukocytosis, and positive blood cultures. 3.) The third syndrome is that of biliary complications, like bile leaks, cholangitis, and strictures causing obstruction. This picture results because the only arterial supply to the allograft's biliary tree is via the hepatic artery[59].

In making the diagnosis of hepatic artery thrombosis, doppler studies have been found to be effective screening studies with a high sensitivity[60]. False negatives do occur and angiogram is frequently needed for confirmation.

At increased risk are small pediatric patients and those requiring complex arterial reconstructions such as double donor arteries and iliac and aortic conduits. In one series of patients[58] double donor arteries had a 24.1% incidence of arterial thrombosis and iliac and aortic conduits had thrombosis rates of 27.7 and 23.1% respectively. Mazzaferro[61] studied 28 variables in pediatric patients and identified five that significantly affected the thrombosis rate. These were diameter $<3$mm, number of revisions, use of iliac and aortic conduits, intraoperative administration of fresh frozen plasma, and post operative anticoagulation.

In our pediatric series, we have had 17 cases of hepatic arterial thrombosis in 16 patients among 123 operations (14%). Because of the variable anatomy, several methods of reconstruction have been employed, and an adaptive strategy is necessary. Our preference is for an aortic Carrel patch to a "branch patch" of the celiac axis of the recipient. Use of the supraceliac aorta of the recipient and avoidance of the infrarenal aorta is also important. Prophylactic use of dextran folowed by aspirin is recommended.

Retransplantation was necessary in 75% of our patients with hepatic artery thrombosis. The indications for retransplantation are acute fulminant hepatic gangrene, inability to control sepsis with antibiotics and drainage, and recurrent cholangitis secondary to strictures. Twenty-five percent of patients with HAT are being managed on chronic antibiotics. Hepatic artery thrombosis remains a dreaded complication with mortalities as high as 50% reported[58].

### PSEUDOANEURYSMS

This is an extremely rare complication of OLT. In a report of eleven pseudoaneurysms, five were nonanastomotic with three of these being related to infection. They were mostly found incidentally on imaging studies, though they may present with rupture, hemobilia or as an enteric fistula. Operative correction is needed because of the high risk of significant complications[62].

### VENOUS COMPLICATIONS

The incidence of portal vein or inferior vena cava thrombosis is much lower than that of arterial thrombosis with rates less than 2% reported[63]. We have had no cases of venous thrombosis at UCLA. The presentation of venous thrombosis depends on the timing of occurrence. Portal vein thrombosis early in the postoperative course presents with severe allograft dysfunction and variceal bleeding if they were preexisting. In cases of late thrombosis, venous collateral have usually developed so that usually there are few allograft problems, and these patients develop varices, ascites and splenomegaly. Similarly, acute caval thrombosis presents with allograft dysfunction, but chronic cases develop insidious onset of ascites, edema, and hepatomegaly. Various imaging studies have been utilized. Hepatobiliary scans generally show delayed excretion in cases of venous thrombosis. Doppler studies are good initial screening tests but have false negatives; angiograms are definitive. Magnetic resonance imaging holds promise, but more experience is needed[64].

One of the major risk factors is pre-existing venous abnormalities in the recipient, and ultrasound to confirm portal vein patency is an important part of a potential recipient's evaluation. In a series of 313 OLT's[65], 16.3% had portal vein, and 2.9% had vena caval abnormalities. The most common was portal vein thrombosis especially in patients who had portosystemic shunts or the Budd Chiari syndrome. Hypoplasia of the portal vein was a finding in some biliary atresia patients with prior portoenterostomies. This has been reported to cause involution of the portal vein[65]. Intraoperative adjustments needed include thrombectomy and/or dissection to the confluence of the splenic and superior mesenteric veins and use of a free graft from the confluence. Caval abnormalities reported include absent retrohepatic cavae in biliary atresia and thrombosis in Budd-Chiari patients. Other contributing factors may be a steal from a large coronary vein shunt and technically imperfect anastomoses.

Treatment of acute portal vein thrombosis include thrombectomy, interposition or jump grafts, or possibly retransplantation if severe graft damaage has occurred. Late thrombosis is treated with portosystemic shunting and/or sclerosis of varices. Patients transplanted for the Budd Chiari syndrome should generally be placed on long term anticoagulation, especially if hypercoagulopathy was the etiology[41].

### BILIARY COMPLICATIONS

Biliary complications used to be the most common and lethal complication following orthotopic liver transplantation, and was considered the "achilles heel" of liver transplantation[66]. With increasing experience and standardization of reconstructions, results have improved significantly. Recent series report biliary tract complications in pediatric patients at 7–20%[56,67].

In adults, biliary leakage is the most common complication, and the most common site is at the T-tube exit site[68]. In children, use of T-tubes are rare and leakage is commonly from the choledochojejunostomy. Presentation is that of fever, abdominal pain, elevated bilirubin and hepatic enzymes, and increased drain output. Diagnosis can be confirmed by transhepatic cholangiogram or ERCP if necessary. Treatment is antibiotic coverage and either simple surgical repair or revision of the anastomosis. Contributing factors to biliary leaks are technical errors at the anastomosis, ischemia from using a too long segment of donor duct,[61] and hepatic artery thrombosis. In our pediatric patients, eight developed biliary leaks, of which five required simple closure and three needed revision of the anastomosis.

Obstruction is the other major biliary complication. The presentation varies from acute cholangitis and liver dysfunction, to recurrent mild fevers and elevated enzymes, to progressive deterioration of allograft function. Etiologies include anastomotic or intrahepatic strictures, T-tube or stent obstruction, stones or recurrent disease like sclerosing cholangitis or cholangiocarcinoma. Diagnosis is confirmed with cholangiogram, or ERCP. Treatment varies with the pathology.

Percutaneous dilatation of a localized stricture has been successful[69], but sometimes operative intervention like revision of a Roux-en-Y, or in cases of multiple intrahepatic strictures, retransplantation may be required.

### NONTECHNICAL HEPATIC COMPLICATIONS

### *Primary Nonfunction*

This occurs when the transplantated hepatic graft, with intact arterial, venous and biliary anastomoses never demonstrates evidence of initial function. The incidence is reported to be between 5–10%[70,71]. The result is a transplanted patient who demonstrates frank hepatic failure with coma, acidosis, massive coagulopathy, renal failure, hypoglycemia and shock. Lab studies confirm the clinical picture, and biopsy reveals profound hepatic necrosis. Patient survival depends on successful retransplantation. Possible causes include an ischemic/preservation injury to the allograft, immunologic injury, or preexisting disease in the donor liver.

### REJECTION

Rejection is the most common cause of allograft dysfunction and indication for retransplantation[68]. It can be classified into acute and chronic rejection. Acute rejection is much more common with mild acute rejection occurring in up to 80% of hepatic allografts. More severe forms occur in 50–75%. The incidence of chronic rejection is generally less than 5%[44].

The presentation of acute rejection is that of fever, abdominal pain, decreased bile production, elevated hepatic enzymes, and decreased synthetic function. More severe cases, or untreated episodes can progress to frank hepatic failure with neurologic and hemodynamic compromise. This presentation is nonspecific and may also be due to technical problems, or various infections.

With no one absolute diagnostic test, the diagnosis is largely clinical and one of exclusion. Acute rejection rarely occurs within the first transplant week. Especially early post-transplant, one must rule out hepatic artery and venous thrombosis with doppler studies. Hepatobiliary scans reflect hepatic function and adequacy of biliary excretion. A thorough workup for infection is also necessary. Frequent use of percutaneous hepatic biopsy is encouraged. It has been shown to be an extremely safe procedure in experienced hands[72] but is not always diagnostic. Findings on biopsy in acute rejection range from mild portal inflammatory infiltrates to severe hepatocyte destruction[73]. Chronic rejection is characterized by the "vanishing bile duct syndrome"[74]. The treatment of rejection is discussed under immunosuppression.

### MISMATCHED GRAFTS

Potential liver donors are matched for size and ABO compatibility. Pre-transplant screening for cytotoxic antibodies are not used clinically. ABO group compatibility is desirable in OLT but not essential for succesful transplantation. Experience at

Pittsburgh[75] and UCLA[44] have demonstrated that results are significantly better in ABO identical grafts vs. ABO nonidentical but compatible grafts, and worst in ABO incompatible grafts. In our pediatric experience 82 ABO identical grafts yielded a survival of 96% while 16 nonidentical but compatible and five incompatible grafts survived 55 and 65% respectively. The use of nonidentical grafts should be reserved for critical situations when no other donor is available.

### NONHEPATIC COMPLICATIONS

### *Infection*

Besides undergoing an extensive surgical procedure, these patients are greatly immunosuppressed, and this combination can expect to yield frequent infectious complications. Major centers have reported infections in 50–80% of patients[44,76]. All classes of organisms are involved with a disproportionately high incidence of viral infections. Kusne, et al[76] reported the infectious complications in 101 consecutive OLT patients. Of these, the overall infection rate was 83% and the percent of patients with bacterial, viral, fungal, and protozoal infections were 59, 53, 18, and 12%, respectively. Important measures include the judicious use of prophylactic antimicrobial agents, frequent surveillance, and aggressive treatment of diagnosed infections.

Prophylactic regimes vary from institutions. At UCLA our prophylactic drugs include broad-spectrum intravenous peri-operative antibiotics for 48 hours, bowel preparation with neomycin and erythromycin base as well as mechanical enemas, and oral nystatin. In patients with biliary atresia and multiple prior abdominal operations, prophylactic amphotericin is used.

Viral infections commonly seen include CMV, HSV and EBV. CMV infections can be serious and a primary cause of mortality. The differentiation from allograft rejection can be difficult as both can present with fever, and elevation in liver enzymes. Patients with CMV hepatitis have been treated and even retransplanted for presumed rejection with fatal results[77]. The liberal use of percutaneous liver biopsies and early treatment is essential. At UCLA we have treated 19 patients with CMV infection with DHPG (gancyclovir) with success in 13.

Risk factors for serious bacterial and fungal infections in liver transplant patients include prolonged intubation and complications related to the hepatic artery or biliary tract. Common severe bacterial infections include intra-abdominal abscesses, pneumonia and cholangitis. Fungal infections have been reported in pediatric patients at a rate of 27–40%[78,53], with the most common organism being candida.

The presence of infections is common in patients dying after OLT. Though a unique cause of death cannot always be established, 13 of 21 of our pediatric deaths had infection as a major contributing factor.

### RENAL COMPLICATIONS

Varying degrees of renal insufficiency are common in liver transplant patients. Many have renal failure pre-transplant from the hepatorenal syndrome.

Post-operative oliguria may be due to hypovolemia, intraoperative hypotension and ATN, sepsis, or nephrotoxic drugs like cyclosporine, aminoglycosides or amphotericin. A study of renal function in our pediatric patients who have been on CsA over one year showed that 85% of the patients had true glomerular filtration rates less than the low normal for age (90cc/min/1.73m2), and there was evidence of progression of impairment with prolonged CsA use[79]. Adequate support of a patient's renal perfusion with volume replacement, and close monitoring of CsA levels are important measures. Patients with severe renal failure may be taken off CsA and immunosuppressed with OKT3.

Volume overload can be managed with use of diuretics, ultrafiltration, or hemodialysis. The need for dialysis has a poor prognosis and the mortality rate of OLT patients requiring dialysis is 40–70%[80].

### GASTROINTESTINAL COMPLICATIONS

Abdominal bleeding manifests with increasing abdominal girth and output from drains. At reoperation, frequently no specific site is found and diffuse oozing is often secondary to coagulopathy[78]. Specific sites may be at vascular anastomoses or the jejunojejunostomy. Other causes of bleeding may be due to stress ulcers or gastritis, varices, herpes esophagitis, or CMV or candida colitis. Endoscopy and biopsies are usually needed for diagnosis. Variceal bleeding after OLT should prompt studies to rule out portal vein thrombosis.

Gastrointestinal perforation occurring early after OLT is frequently due to unrecognized intra-operative injury. The frequency is much higher in patients who have had previous intraabdominal surgery and adhesions. Injuries like denuded serosa and small electrocautery burns can progress to full thickness perforation. Gastrointestinal leaks at the jejunojejunostomy can also occur due to technical errors. Patients present with intraabdominal sepsis and free intra-abdominal air, and operative repair is necessary. Late perforation is less common and etiologies include perforation secondary to lymphoproliferative disease[81] or infectious ulcers.

GI obstruction is usually due to post-operative adhesions. Rarer causes include herniation of the jejunojejunostomy or lymphoproliferative diseases.

Mild transient hyperamylasia is common in OLT patients and usually no treatment is necessary. Significant pancreatitis is rare. Patients at increased risk are those who have undergone extensive peripancreatic dissection like with portal vein grafts, and one study found an increased incidence in HBsAg + patients[82].

### PULMONARY COMPLICATIONS

Some form of pulmonary morbidity occurs in essentially all liver transplant recipients. Atelectasis, effusions, and infection are the most common. Atelectasis usually resolves with aggressive pulmonary toilet and as patients get more ambulatory. Twenty percent required bronchoscopy in one series[78]. Right pleural effusions are also very common and usually resolve over time. Thoracentesis or thoracostomy is necessary in 15–20% of patients due to large symptomatic effusions or suspicion of infection. Paralysis of the right hemidiaphragm is seen after OLT and is felt to be due to an intra-operative injury to the phrenic nerve when the superior vena caval clamp is applied.

### NEUROLOGIC COMPLICATIONS

One of the more common complications after OLT is seizures. The incidence is up to 20%[44]. Many are related to high CsA and low magnesium levels. Patients usually have normal CT scans and lumbar punctures and recover without residual deficits.

Other complications include meningitis, extrapyramidyl symptoms, and paresthesias. Positioning injuries like brachial plexus and peroneal nerve injuries are also seen. Intracranial hemorrhage occurs in patients with severe liver failure and is a frequent lethal event in these patients.

### IMMUNOSUPPRESSION

Improvements in our ability to prevent and treat rejection is a critical factor in the development and success of solid organ transplantation. Experience with many of the drugs in use was initially gained in human kidney transplantation. In the early 1960's, the use of azathioprine opened the way for the development of clinical organ transplantation. The early liver transplants received double-drug therapy with azathioprine and corticosteroids with limited success. In the mid-1960's, results improved somewhat when triple-drug therapy was introduced with the addition of antilymphocyte globulin (ALG). The most dramatic improvements occurred with the clinical use of cyclosporine A in the early 1980's which introduced a new era in organ transplantation[83]. Currently, we utilize glucocorticoids, azathioprine, CsA, and the monoclonal anti-T cell antibody OKT3.

### GLUCOCORTICOIDS

Glucocorticoids have been an essential part of transplant immunosuppression. They are used universally in induction therapy in combination with other drugs, and also for the treatment of allograft rejection. Steroids have multiple anti-inflammatory and immunologic effects that have been described extensively. The mechanism by which glucocorticoids inhibit allograft rejection is by the inhibition of interleukin I and interleukin II, and therefore the stimulation of cytotoxic T-cells[84].

Side effects are well known and include increased susceptibility to infection, growth retardation, gastrointestinal ulcers, fluid retention, truncal obesity and osteoporosis. The dose of steroids has been able to be decreased with multiple drug regimens in attempts to minimize the toxicities.

### AZATHIOPRINE

Azathioprine or imuran is used in induction therapy with steroids and CsA. It is a derivative of 6-mercaptopurine and functions as an antimetabolite of purine synthesis. It therefore is most effective against rapidly dividing cells[85]. Azathioprine dosing is between 1–4 mg/kg/day. Major side effects are neutropenia and thrombocytopenia which may limit its use in some cases[86]. Other toxicities include increased risk of infection, anemia, and possibly pancreatitis and hepatitis.

### CYCLOSPORINE A

The introduction of CsA into clinical transplantation has revolutionized the field. It is a much more useful drug than any of its precursors due to its potent immunosuppressive actions and relatively less toxicities. CsA works by interfering with the action of interleukin 2 which consequently blocks activation of cytotoxic T cells while T suppressor cells continue to function[87]. It is a lipophyllic drug that can be administered orally or intravenously. It is metabolized by the hepatic P450 enzyme system, and other drugs which affect the P450 enzymes will influence CsA metabolism. CsA levels are monitored via high pressure liquid chromatography (HPLC) techniques which measures levels of only the parent compound, or less specific radioimmunoassay (RIA) tests which measure the less active metabolites also.

CsA is far from being the ideal immunosuppressant due to its toxicities. The most important of these is its nephrotoxicity. It is often reversible, but long lasting impairment also occurs (see Renal Failure). It is important to establish adequate urine output post-OLT before administering CsA, and carefully monitoring levels. Other side effects include hepatotoxicity with hyperbilirubinemia, hypertension, sodium and fluid retention, magnesium wasting, seizures, and hirsutism.

### OKT3

OKT3 is a murine monoclonal antibody that binds to the T3 protein located on the surface of all peripheral T cells[88]. This binding blocks the activation of the T cells and also facilitates opsonization of the bound cells. It was first shown to be effective in reversing acute renal allograft rejection[89] and since then has been used in liver transplantation. The UCLA experience with using OKT3 to treat steroid-resistant rejection was reported in 1987[90]. In our pediatric patients, OKT3 has been used to treat 43 episodes of steroid-resistant rejection in 31 patients with a 79% success rate. Dosing of OKT3 is adjusted by measuring CD3+ cells in peripheral blood.

Toxicities of OKT3 include increased risk of infections, especially viral, pulmonary edema, flu-like reactions with fever and chills and a meningitis syndrome. Premedication with steroids and acetaminophen for the initial doses helps decrease the flu-like reactions. One should also assure that the patient is clinically euvolemic with a clear chest X-ray to decrease the risk of pulmonary edema which can be lethal. If necessary, patients should be diuresed or dialyzed before the initial dose.

### FK 506

FK506 is a recently introduced macrolide compound isolated in Japan from a soil fungus, *Streptomyces tsukubanesis* that has been shown to have potent immunosuppressive effects[91]. Its exact mechanism of action has not been completely elucidated, but it appears to share similarities with cyclosporine A in that a major effect of both is inhibition of interleukin 2 (IL2) production. However, FK 506 is up to 100× more potent in inhibiting IL2 mRNA production in activated human peripheral blood T-cells than cyclosporine[92].

The potent immunosuppressive effects of FK506 have been confirmed in various animal models including rat heart allografts, kidney and liver transplants in dogs[93,94], as well as kidney transplants in baboons[95]. Much excitement has been generated about the use of FK506 clinically because of its remarkable potency at low doses and apparant lack of significant toxicities. Initial human trials have begun and a large multicenter trial will start shortly.

### CURRENT UCLA IMMUNOSUPPRESSION PROTOCOL

### *Induction Therapy*

We employ triple-drug therapy with steroids, cyclosporine A, and azathioprine. Methylprednisolone is started intravenously at the time of transplant at 20–30mg/kg and rapidly tapered to .3–5mg/kg/day over the first week. When oral intake resumes, the steroids are given as oral prednisone at an equivalent dose.

The first CsA dose is given pre-operatively at 15mg/kg orally. The post-operative doses are started after adequate urine output is established at 3–5mg/kg/day intravenously in two divided doses. Each dose is infused over four hours. The dose is adjusted daily based on CsA trough plasma levels with a target range of 200–300ng/ml. After oral intake is established, oral CsA is started at 10–15mg/kg/day in two divided doses. The intravenous dose is continued and tapered as therapeutic levels are reached with the oral dose. The overlap is usually 5–7 days. In cases of renal insufficiency, CsA doses are decreased accordingly or even held as indicated.

Azathioprine is started at 1–2mg/kg/day intravenously on the first post-transplant day and later converted to the same oral dose. It is held in cases of neutropenia or severe sepsis.

### MAINTENANCE IMMUNOSUPPRESSION

Again, triple-drug therapy is used in most patients. Prednisone is tapered gradually and maintained on as low a dose as possible. Cyclosporine doses are adjusted so that higher levels are maintained early after OLT but allowed to drop to the 140–160ng/ml range in stable patients greater than one year after OLT. In patients with stable levels and renal function, levels are checked about every two months. Azathioprine is maintained at 1mg/kg/day.

### TREATMENT OF REJECTION

Patients diagnosed with rejection are initially treated with intravenous methylprednisolone at 20mg/kg with tapering over five days to the pre-treatment dose. Patients not responding or having recurrent rejection are generally rebolused if no other cause of allograft dysfunction is apparent. Patients felt to have steroid-resistant rejection are currently treated with OKT3. The intravenous dose is 2.5mg for children <30kg. or 5mg for those >30kg. A typical treatment course lasts 10–14 days. If peripheral T3+ cells exceeds 10%, the dose of OKT3 is increased. Persistent refractory rejection is either treated with antilymphocyte globulin or retransplantation.

### RESULTS

The survival of pediatric patients after OLT has shown progressive improvement over time. Major factors have been improved immunosuppression especially with cyclosporine, refinement of operative techniques and increasing technical experience, earlier referral and selection of patients for OLT, and improvements in perioperative management. One year survival prior to 1980 were 30–40%, but with the above mentioned improvements, current pediatric survival rates exceed 70% in most centers. Recent pediatric series report two and three year survivals of 86[96] and 73%[78]. The five year actuarial survival curve for the first 123 pediatric OLTs performed at UCLA is shown in Figure 1. The overall 5 year survival is 77%. The prognosis is better for metabolic diseases than tumors or biliary atresia. The majority of deaths occurred within 5 months, so the survival curves can be expected to be maintained.

Besides survival, the success of a procedure on children depends on the successful growth and development of the transplant recipients. Multiple reports have documented the satisfactory physical and intellectual development in the majority of patients. In a UCLA study of 19 patients, 16 exhibited normal or accelerated growth after transplantation[97]. In the Pittsburgh pediatric series reported in 1987[98], 50% achieved accelerated growth, and 45 of 57 children were in an age-appropriate school grade or only one year behind. Essentially all our current school aged survivors are able to attend school.

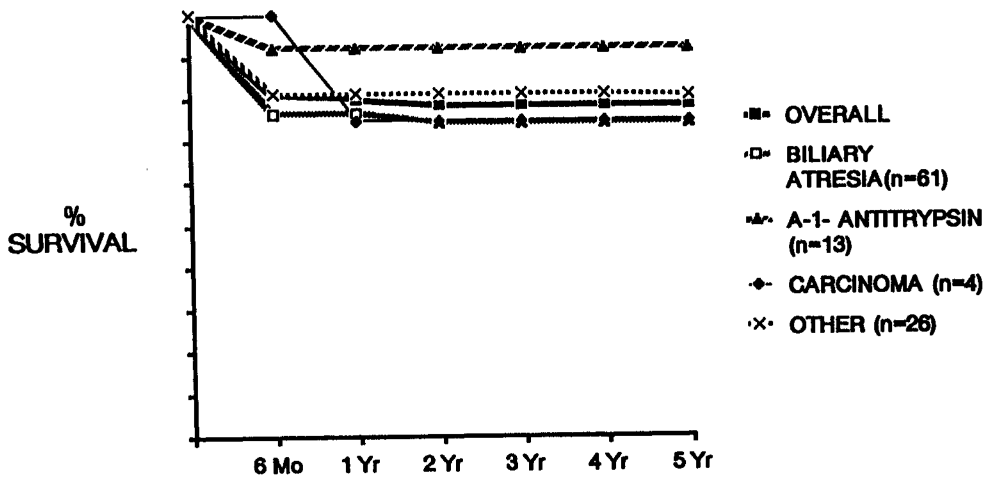

FIGURE 1. Actuarial survival curves by diagnosis. Overall 5-year actuarial survival of 77%. Kaplan-Meier method.

### CONCLUSION

Liver transplantation is able to cure children with end-stage diseases with acceptable morbidity and mortality, and improve their quality of life. Results can expect to improve with greater availability of donor organs, earlier referral of candidates to transplant centers, further technical refinements, and the development of more specific, less toxic immunosuppressive agents.

### *References*

1. NIH Consensus Development Conference Statement: (1983) Liver transplantation. June 20–23, *Hepatology* (1984) **4**, 1075–1095
2. Starzl, T.E., Marchiaro, T.L., Von Kaulla, K. *et al*: (1963) Homotransplantation of the liver in humans. *Surg. Gynecol. Obstet.* **117**, 659–676
3. Esquivel, C.O., Iwatsuki, S. and Gordon, R.D., *et al*: (1987) Indications for pediatric liver transplantation. *The Journal of Pediatrics* **111**, 1039–1045
4. Alagille, D. (1984) Extrahepatic biliary atresia. *Hepatology* **4**, 7S–10S.
5. Kasai, M. and Suzuki, S. (1959) A new operation for "noncorrectable" biliary atresia: hepatic portoenterostomy. *Shujutsu* **13** 733–739
6. Kasai, M., Kimura, S. and Asakura, Y. *et al*: (1968) Surgical treatment of biliary atresia. *Journal of Pediatric Surgery* **3**, 665–675
7. Barkin, R.M. and Lilly, J.R. (1980) Bilary atresia and the Kasai operation: Continuing care. *J Pediatr.* **96**, 1015–1019
8. Suruga, K., Miyano, T. and Kitahara, T. *et al*: (1981) Treatment of biliary atresia: a study of our operative results. *J. Pediatr. Surg.* **16**, 896–898
9. Ito, T., Horisawa, M. and Ando, H. (1983) Intrahepatic bile ducts in biliary atresia: A possible factor in determining prognosis. *J. Pediatr. Surg.* **18**, 124
10. Ohi, R., Hanamatsu, M. and Mochizuki, I. *et al*: (1985) Progress in the treatment of biliary atresia. *World J. of Surg.* **9**, 285–293
11. Ascher, N.L. and Najarian, J.S. (1984) Hepatic transplantation and biliary atresia: Early experience in eight patients. *World. J. of Surg.* **8**, 57–63
12. Pettit, B.j., Zitelli, B.J. and Rowe, M.I. (1984) Analysis of patients with biliary atresia coming to liver transplantation. *J. Pediatr. Surg.* **19**, 770–785
13. Altman, R.P. (1979) Results of reoperation for correction of extrahepatic biliary atresia. *J. Pediatr. Surg.* **14**, 305–309
14. Millis, J.M., Brems, J.J. and Hiatt, J.R. *et al*: (1988) Orthotopic liver transplantation for biliary atresia. *Arch. Surg.* **123**, 1237–1239
15. Sharp, H.L., Bridges, R.A. and Krivit, W. *et al*: (1969) Cirrhosis associated with alpha-1-antitrypsin deficiency: A previously unrecognized inherited disorder. *J. Lab. and Clin. Med.* **73**, 934–939
16. Erickson, S.L. (1965) Studies in alpha-1-antitrypsin. *Acta. Med. Scand.*(Suppl) **423**, 177
17. Sharp, H.L. (1976) The current status of A-1-antitrypsin, a protease inhibitor, in gastrointestinal disease. *Gastroenterology* **70**, 611–621
18. Sveger, T. (1976) Liver disease in alpha-1-antitrypsin deficiency detected by screening of 200,000 infants. *N. Engl. J. Med.* **194**, 1316–1321
19. Sharp, H.L. (1971) Alpha-1-antrypsin deficiency. *Hosp. Prac.* May 83–96
20. Alagille, D. (1984) Alpha-1-antitrypsin deficiency. *Hepatology* **4**, 11S–14S
21. Hood, J.M., Koep, L.J. and Peters, R.L. *et al*: (1980) Liver transplantation for advanced liver disease with alpha-1-antitrypsin deficiency. *N. Eng. J. Med.* **302**, 272–275
22. Esquivel, C.O., Vincente, E. and Van Thiel, R. *et al*: (1987) Orthotopic liver transplantation for alpha-1-antitrypsin deficiency: an experience in 29 children and 10 adults. *Trans. Proc.* **19**, 3798–3802
23. Carson, N.A.J., Biggart, J.D. and Bitles, A.H. *et al*: (1976)Hereditary tyrosinemia: Clinical, enzymatic, and pathological study of an infant with the acute form of the disease. *Arch. Dis. Child,* **51**, 106

24. Starzl, T.E., Zitelli, B.J. and Shaw, B.W. Jr. *et al*: (1985) Changing concepts: Liver replacement for hereditary tyrosinemia and hepatoma. *J. Ped.* **106**, 604–606
25. Scheinberg, I.H. and Sternlieb, I. (1984) Wilson's disease. Philadelphia: W.B. Saunders, 9–16
26. Sternlieb, I. (1984) Wilson's disease: indications for liver transplants. *Hepatology* **4**, 15S–17S
27. Walshe, J.M. (1973) Copper chelation in patients with Wilson's disease. *QJ Med* **42**, 441–452
28. McCullough, A.J., Fleming, C.R. and Thistle, J.L. *et al*: (1983) Diagnosis of Wilson's disease presenting as fulminant hepatic failure. *Gastroenterology* **84**, 101–107
29. Crigler, J.F. and Najjar, V.A. (1952) congenital familial nonhemolytic jaundice with kernicterus. *Pediatrics* **10**, 169–180
30. Bloomer, J.R. and Sharp, H.L. (1984) The liver in Crigler-Najjar Syndrome, Protoporphyria, and other metabolic disorders. *Hepatology* **4**, 18S–21S
31. Bloomer, J.R. (1982) Protoprophyria. *Seminars in Liver Disease* **2**, 143–153
32. Fyle, M.W. and Jendrisak, M.D. (1986) Liver transplantation in the child. *World J. Surg.* **10**, 432–441
33. Trey, C. andDavidson, L.S. (1970) in Popper H, Schaffner F(eds.): Progress in Liver Diseases. New York, Grune and Stratton, p282
34. European Association for the Study of the Liver. (1979) Randomized trial of steroid therapy in acute liver failure. *Gut* **20**, 620–623
35. Christensen, E., Bremmelgoard, A. and Tygstrup, N. *et al*: (1983) Prediction of fatality in fulminant hepatic failure. *Scand. Gastroenterol* **19**, 90–96
36. Ring-Larsen, H. and Palazzo, V. (1981) Renal failure in fulminant hepatic failure and terminal cirrhosis. *Gut* **22**, 585--591
37. Brems, J.J., Hiatt, J.R. and Ramming, K.P. *et al*: (1987) Fulminant hepatic failure: the role of liver transplantation as primary therapy. *Am. J. Surg.* **154**, 137–141
38. Sherlock, D.S. (1985) Diseases of the liver and biliary system ed 7. London, Blackwell Scientific Publications
39. Ahn, S., Yellin, A. and Sheng, F.C. *et al*: (1987) Selective surgical therapy of the Budd-Chiari syndrome provides superior survival rates than conservative management. *J. Vasc. Surg.* **5**, 28–37.
40. Scharschmidt, B.R. (1984) Human liver transplantation: analysis of data of 540 patients from four centers. *Hepatology* **4**, 95–101
41. Campbell, D.A., Jr. Rolles, K. and Jamieson, N. *et al*: (1988) Hepatic transplantation with perioperative and long term anticoagulation as treatment for Budd-Chiari syndrome. *Surg. Gyne. Obs.* **166**, 511–518
42. Robbins, S.L., Cotran, R.S., and Kumar, V. (1984) Pathologic basis of disease, ed 3. W.B. Sanders Co.
43. Demetris, A.J., Joffe, R. and Sheahan, D.G. *et al*: (1986) Recurrent hepatitis B in liver allograft recipients. *Am. J. Pathol.* **125**, 161–172
44. Busuttil, R.W., Colonna, J.O. and Hiatt, J.R. *et al*: (1987) The first 100 liver transplants at UCLA. *Ann. Surg.* **206**, 387–402
45. Starzl, T.E., Iwatsuki, S. and Shaw, B.W. Jr, *et al*: (1986) Treatment of fibrolamellar hepatoma with partial hepatectomy or with total hepatectomy and liver transplantation. *Surg. Gynecol. Obstet.* **162**, 145–148
46. Koneru, B., Flye, M.W. and Busuttil, R.W. *et al*: Liver Transplantation for Hepatoblatoma—The American Experience. *Ann. Surg.*. Submitted for publication
47. Busuttil, R.W., Seu, P. and Millis, J.M. *et al*: Liver Transplantation in Children. *Ann. Surg.*. Accepted for publication
48. Quinones-Baldrich, W.J., Memsic, L. and Ramming, K. *et al*: (1986) Branch patch for arterialization of hepatic grafts. *Surg. Gynecol. Obstet* **162**, 488–499
49. Fortner, J.G., Yeh, S.D., and Kim, D.K. *et al*: (1979) The case for and technique of heterotopic liver grafting. *Trans. Proc.* **11**, 269
50. Shaw, B.W., Wood, R.P. and Kaufman, S.S. *et al*: (1988) Liver transplantation therapy for children: Part I. *J. Ped. Gastroenterol. Nutr.* **7**, 157–166
51. Bismuth, H. and Houssin, D. (1984) Reduced-sized orthotopic liver graft in hepatic transplantation in children. *Surgery* **95**, 367
52. Broelsch, C.E., Emond, J.C. and Thistlewaite, J.R. *et al*: (1988) Liver Transplantation, including the concept of reduced-size liver transplants in children. *Ann. Surg.* **208**, 410–420
53. Otte, J.B., de Ville de Goyet, J. and Solak, E. *et al*: (1990) Size Reduction of the donor liver is a safe way to alleviate the shortage of size-matched Organs in Pediatric Liver Transplantation. *Ann. Surg.* **211**, 146–157

54. Kalayooglu, M., Sollinger, H.W. and Stratta, R.J. *et al*: (1988) Extended preservation of the liver for clinical transplantation. Lancet; 617–619
55. Belzer, F.O., Sollinger, H.W. and Glass, N.R. *et al*: (1984) Beneficial effects of adenosine and phosphate in kidney reservation. *Transplant Proc.* **16**, 161–163
56. Kalayoglu, M., Stratta, R.J. and Sollinger, H.W. *et al*: (1989) Liver Transplantation in infants and children. *J. Ped. Surg.* **24**, 70–76
57. Hoffer, F.A., Teele, R.L. and Lillehei, C.W. *et al*: (1988) Infected bilomas and hepatic artery thrombosis in infant recipients of liver transplants. *Radiology* **169**, 435–438
58. Tzakis, A.G., Gordon, R.D. and Shaw, B.W. *et al*: (1985) Clinical presentation of hepatic artery thrombosis after liver transplantation in the cyclosporine era. *Transplantation* **40**, 667–671
59. Northover, J. and Terblanch, J. (1978) Bile duct blood supply. Its importance in human liver transplantation. *Transplantation* **26**, 67–69
60. Penkrot, R.J. (1986) Noninvasive evaluation of complications of orthotopic liver transplantation. *Semin. Intervent. Radiol.* **3**, 120–122
61. Mazzaferro, V., Esquivel, C.O. and Makowka, L. *et al*: (1989) Hepatic artery thrombosis after pediatric liver transplantation—a medical or surgical event? *Transplantation* **47**, 971-977
62. Tobben, P.J., Zajko, A.B. and Sumkin, J.H. *et al*: (1988) Pseudoaneurysms complicating organ transplantation: roles of CT, duplex sonography, and angiography, *Radiology* **169**, 65–70
63. Koneru, B., Tzakis, A.G. and Bowman, J. *et al*: (1988) Postoperative surgical complications. *Gastro. Clinics North Am.* **17**, 71–91
64. Day, D.L., Letourneau, J.G. and Allan, B.T. *et al*: (1986) MRI evaluation of upper abdominal vascular anatomy in pediatric liver transplant candidates. *AJR* **147**, 1027–1030
65. Lerut, J., Tzakis, A.G. and Bron, K. *et al*: (1987) Complications of venous reconstruction in human orthotopic liver transplantation. *Ann. Surg.* **205**, 404–414
66. Calne, R.Y. (1976) A new technique for biliary drainage in orthotopic liver transplantation utilizing the gallbladder as a pedicle graft conduit between the donor and recipient common bile ducts. *Ann. Surg.* **184**, 605–609
67. Stratta, R.J., Wood, P. and Langnas, A.N. *et al*: (1989) Diagnosis and treatment of biliary tract complications after orthotopic transplantation. *Surgery* **106**, 675–684
68. Lerut, J., Gordon, R.D., Iwatsuki, S. and Starzl, T.E. (1987) Surgical complications in human orthotopic liver transplantation. *Acta. Chir. Belg.* **87**, 193–204
69. Zajko, A.B., Campbell, W.L. and Bron, K.M. *et al*: (1985) Cholangiography and interventional biliary radiology in adult liver transplantation. *Am. J. Radiol.* **144**, 127–133
70. Shaw. B.W. Jr., Gordon, R.D. and Iwatsuki, S. *et al*: (1985) Hepatic retransplantation. *Trans. Proc.* **17**, 264–271
71. Miller, C., Mazzaferro, V. and Makowka, L. *et al*: (1988) Rapid flush technique for donor hepatectomy: safety and efficacy of an improved method of liver recovery for transplantation. *Transplant Proc.* **20**, 948
72. Perrault, J., McGill, D.G. and Ott, B.J. *et al*: (1978) Liver biopsy: Complications in 1000 inpatients and outpatients. *Gastroenterology* **74**, 103–106
73. Ray, R.A., Lewin, K.J. and Colonna, J. *et al*: (1988) The role of liver biopsy in evaluating acute alograft dysfunction folowing liver transplantation: a clinical histologic correlation of 34 liver transplants. *Human Pathol.* **19**, 835–848
74. Calne, R.Y. and Williams, R. (1977) Orthotopic liver transplantation: The first 60 patients. *Br. Med. J.* **1**, 471–476
75. Gordon, R.D., Iwatsuki, S. and Esquivel, C.O. *et al*: (1986) Liver transplantation across ABO blood groups. *Surgery* **100**, 342–348
76. Kusne, S., Dummer, J.S. and Singh, N. *et al*: (1988) Infections after Liver Transplantation. An analysis of 101 consecutive cases. *Medicine* **67**, 132–143
77. Bronsther, O., Makowka, L. and Jaffe, R. *et al*: (1988) Occurrence of Cytomegalovirus Hepatitis in Liver Transplant Patients. *J. Medic. Virol.* **24**, 423–434
78. Andrews, W.S., Wanek, E. and Fyock, B. *et al*: (1989) Pediatric liver transplantation: A 3-year experience. *J. Pediatr. Surg.* **24**, 77–82
79. McDiarmid, S. Ettenger, R. and Hawkins, R. *et al*: (1990) The Impairment of True GFR in long-term CsA treated pediatric allograft recipients. Transplantation; in press
80. Wood, R.P., Shaw, B.W. and Starzl, T.E. *et al*: (1985) Extrahepatic complications of liver transplantation. *Seminars in liver disease* **5**, 377–384
81. Makowka, L., Malesnik, M. and Stieber, A. *et al*: (1987) Control of posttransplant lymphoproliferative disorders and Kaposi's sarcoma by modulation of immunosuppression. In Good RA,

Schattauer FK (eds): The Nature, Cellular and Biochemical Basis and Management of Immunodeficiencies. New York, Verlag, Stuttgart
82. Alexander, J.A., Demetrius, A.J. and Gavaler, J.S. *et al*: (1988) Pancreatitis following liver transplantation. *Transplantation* **45**, 1062–1065
83. Starzl, T.E., Iwatsuki, J. and Van Thiel, D.H. *et al*: (1982) Evaluation of liver transplantation. *Hepatology* **2**, 614--636
84. Giles, S., Crabtree, G.R. and Smith, G.A. (1979) Glucocorticoid induced inhibition of T cell growth factor production. *J. Immunol.* **123**, 1624
85. Elion, G.B., Callahan, S. and Bieber, S. *et al*: (1961) A summary of investigations with 2-amino-6-methy-4-nitro-5-imidazolyo-thiopurine. *Cancer Chemotherap. Rep.* **14**, 93
86. Haessleim, H.C., Pierce, V.C. and Lee, H.M. *et al*: (1972) Leukopenia and azathioprine management. *Renal Homotranspsl* **71**, 598
87. Hess, A.D., Tutschka, R.J. and Santos, G.W. *et al*: (1982) Effect of cyclosporine A on human lymphocyte responses in vitro. *J. Immunol.* **128**, 355
88. Terhorst, C. (1986) Structure and function of the T-cell receptor/T3 complex. *Transplant Proc.* **18**, 931–936
89. Goldstein, G., Schindler, J. and Tsai, H. *et al*: (1985) A randomized clinical trial of OKT3 monoclonal antibody for acute rejection of cadaveric renal transplants. *N. Engl. Med.* **313**, 337–342
90. Colonna, J.O., Goldstein, L.I. and Brems, J.J. *et al*: (1987) A prospective study on the use of monoclonal anti-T3-cell antibody (OKT3) to treat steroid-resistant liver transplant rejection. *Arch. Surg.* **122**, 1120–1123
91. Kino, T., Hatanaka, H. and Hashimoto, M. *et al*: (1987) FK 506, a novel immunosuppressant isolated from a Streptomycetes. I. Fermentation, isolation, and physio-chemical and biological characteristics. *J. Antibiot.* **60**, 1249
92. Tocci, M.J., Matkovich, D.A., Collier, K.A. *et al.*: (1989) The immunosuppressant FK506 selectively inhibits expression of early T cell activation genes. **143**, 718–726
93. Ochiai, T., Nakajima, K. and Nagata, M. *et al*: (1987) Studies of the induction and maintenance of long-term graft acceptance by treatment with FK506 in heterotopic cardiac allotransplantation in rats. *Transpl.* **44**, 734–738
94. Todo, S., Murase, N. and Ueda, Y. *et al*: (1988) Effect of FK506 in experimental organ transplantation. *Transpl. Proceedings* **20**, 215–219
95. Starzl, T.E., Todo, S. and Tzakis, A.G. *et al*: (1989) Liver transplantation: An unfinished product. *Transpl. Proceedings* **21**, 2197–2200
96. Otte, J.B., Yandza, T. and de Ville de Goyet, J. *et al*: (1988) Pediatric liver transplantation: Report on 52 patients with a 2-year survival of 86% *J. Pediatr. Surg.* **3**, 250–253
97. Spolidoro, J.V., Pehlivanoglu, E. and Berquist, W.E. *et al*: (1987) Growth acceleration in children post orthotopic liver transplantation (OLT). *Clin. Res.* **35**(1), 209A
98. Zitelli, B.J., Gartner, J.C. and Malatack, J.J. *et al*: (1987) Pediatric liver transplantation. *Transpl. Proc.* **19**, 3309–3316

*(Accepted by S. Bengmark 23 April 1990)*

